# A double-blind, randomized controlled trial to compare the effect of biannual peripheral magnetic resonance imaging, radiography and standard of care disease progression monitoring on pharmacotherapeutic escalation in rheumatoid and undifferentiated inflammatory arthritis: study protocol for a randomized controlled trial

**DOI:** 10.1186/1745-6215-15-268

**Published:** 2014-07-05

**Authors:** Ruben Tavares, Karen Anne Beattie, William George Bensen, Raja S Bobba, Alfred A Cividino, Karen Finlay, Ron Goeree, Lawrence Errol Hart, Erik Jurriaans, Maggie J Larche, Naveen Parasu, Jean-Eric Tarride, Colin E Webber, Jonathan D Adachi

**Affiliations:** 1 UNCOVER Clinical Research Company, Milton, ON, Canada; 2 Department of Medicine, Division of Rheumatology, McMaster University, Hamilton, ON, Canada; 3 Department of Diagnostic Imaging, Juravinski Hospital, Hamilton, ON, Canada; 4 Department of Medicine, Division of Radiology, McMaster University, Hamilton, ON, Canada; 5 Department of Clinical Epidemiology and Biostatistics, McMaster University, Hamilton, ON, Canada; 6 Programs for Assessment of Technology in Health (PATH) Research Institute, St Joseph’s Healthcare Hamilton, Hamilton, ON, Canada; 7 Department of Economics, McMaster University, Hamilton, ON, Canada; 8 Department of Nuclear Medicine, Hamilton Health Sciences, Hamilton, ON, Canada

**Keywords:** Arthritis, Rheumatoid, Randomized control trial, Magnetic resonance imaging, Radiography, Antirheumatic agents, Disease management

## Abstract

**Background:**

Permanent joint damage is a major consequence of rheumatoid arthritis (RA), the most common and destructive form of inflammatory arthritis. In aggressive disease, joint damage can occur within 6 months from symptom onset. Early, intensive treatment with conventional and biologic disease-modifying anti-rheumatic drugs (DMARDs) can delay the onset and progression of joint damage. The primary objective of the study is to investigate the value of magnetic resonance imaging (MRI) or radiography (X-ray) over standard of care as tools to guide DMARD treatment decision-making by rheumatologists for the care of RA.

**Methods:**

A double-blind, randomized controlled trial has been designed. Rheumatoid and undifferentiated inflammatory arthritis patients will undergo an MRI and X-ray assessment every 6 months. Baseline adaptive randomization will be used to allocate participants to MRI, X-ray, or sham-intervention groups on a background of standard of care. Prognostic markers, treating physician, and baseline DMARD therapy will be used as intervention allocation parameters. The outcome measures in rheumatology RA MRI score and the van der Heijde-modified Sharp score will be used to evaluate the MRI and X-ray images, respectively. Radiologists will score anonymized images for all patients regardless of intervention allocation. Disease progression will be determined based on the study-specific, inter-rater smallest detectable difference. Allocation-dependent, intervention-concealed reports of positive or negative disease progression will be reported to the treating rheumatologist. Negative reports will be delivered for the sham-intervention group. Study-based radiology clinical reports will be provided to the treating rheumatologists for extra-study X-ray requisitions to limit patient radiation exposure as part of diagnostic imaging standard of care. DMARD treatment dose escalation and therapy changes will be measured to evaluate the primary objective. A sample size of 186 (62 per group) patients will be required to determine a 36% difference in pharmacological treatment escalation between the three groups with intermediate dispersion of data with 90% power at a 5% level of significance.

**Discussion:**

This study will determine if monitoring RA and undifferentiated inflammatory arthritis patients using MRI and X-ray every 6 months over 2 years provides incremental evidence over standard of care to influence pharmacotherapeutic decision-making and ultimately hinder disease progression.

**Trial registration:**

This trial has been registered at ClinicalTrials.gov: NCT00808496 (registered on 12 December 2008).

## Background

Inflammatory arthritis (IA) affects up to 3% of the adult population [1]. It is comprised of the following specific diagnoses: ankylosing spondylitis, psoriatic arthritis, reactive arthritis, rheumatoid arthritis (RA), inflammatory osteoarthritis and undifferentiated IA (UIA). Of these forms, RA is most common with a prevalence ranging from 0.5 to 2.0% with a commonly accepted figure of 1% [2,3].

Early in the disease course, RA results in pain, joint swelling and stiffness, fatigue, and functional disability [3-5]. In the long term, the majority of RA patients suffer permanent erosive joint damage, increased risks for cardiovascular and systemic comorbidities, and a higher mortality rate [6-8]. Joint destruction begins early in the disease course [9-13]. Within 3 years from symptom onset, up to 90% of patients develop irreversible joint damage [12,13]. Various studies have demonstrated that magnetic resonance imaging (MRI) is more sensitive than radiography (X-ray) at detecting early articular changes leading to and including erosive joint damage [14-17].

Erosive disease on X-ray is the classic hallmark of longstanding, destructive RA [18,19]. The second notable X-ray prognostic feature is joint space narrowing (JSN) [20,21]. Although erosions have been linked to physical deformity and long-term functional declines [22], more recent data suggest that JSN may have a greater relative effect on these outcomes in disease subsets [23].

In contrast, RA pathological features measured on MRI using the Outcomes in Rheumatology (OMERACT) RA MRI Score (RAMRIS) include synovitis, bone marrow edema, and erosions [24]. Synovitis is a precursor to bone marrow edema [25] and both predict erosions [26-28]. Cross-sectionally, erosions on MRI do not correlate well with X-ray erosions [29,30]. Erosions on MRI may represent bone lesions at an earlier stage of development below the lower limit of detection of X-ray. With time, MRI lesions may increase in size to be detected on X-ray [30-32]. Early in the development of OMERACT RAMRIS the decision was made not to measure other notable features including, but not limited to, tenosynovitis and JSN [33].

Limiting disease progression on diagnostic imaging remains an important clinical and regulatory outcome [34]. Early pharmacologic treatment of RA with conventional and biologic disease-modifying anti-rheumatic drugs (DMARDs) has proven to improve clinical [35-38] and radiological [38] outcomes. Treat-to-target strategies of maintaining low disease activity using anti-rheumatic drug augmentation have also been demonstrated to improve clinical outcomes in patients [39]. Despite optimal control of clinical and laboratory findings, erosive disease progression may continue to progress for subsets of the disease population [17]. These data suggest that diagnostic imaging may complement pharmacotherapeutic decision-making. However, diagnostic imaging is infrequently and unsystematically applied to routine disease management [40]. Disease monitoring with sensitive and responsive imaging modalities may help to augment therapy and further delay disease progression.

The primary objective of the study is to investigate the relative value of MRI or X-ray over standard of care as tools to guide DMARD treatment decision-making by rheumatologists for the care of RA and UIA. Specifically, the study compares the effect of biannual monitoring of disease progression with: 1) 1.0 T extremity MRI of the second to fifth metacarpophalangeal joints (MCPs) of the worst-affected hand at baseline; 2) X-rays of both hands and wrists; and 3) standard of care on the rate of augmenting DMARD treatment decision-making over 2 years. The co-primary objective aims to determine differences in diagnostic imaging evidence of disease progression with biannual monitoring using MRI or X-ray over standard of care over 2 years.

Secondary, exploratory objectives include the following. The effect of intervention-based pharmacotherapeutic augmentation on diagnostic imaging evidence of disease progression, composite measures of disease activity, function, and quality of life will be determined. The difference in the frequency of changes detected between the two active interventions will be determined. The effect of study participation bias on the standard of care frequency of diagnostic imaging usage in disease management of the population will be determined. Healthcare resource utilization (for example, number of physician visits, hospitalizations) and productivity losses for this population and across study groups will be investigated. Blinded, biannual in-term assessments of these outcomes will also be investigated.

## Methods/design

### Study design

**Figure 1:**
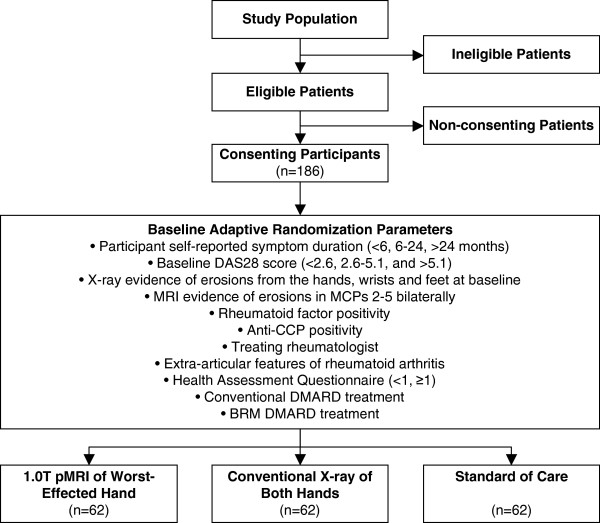
**Study participant flow diagram and intervention allocation prognostic variables.** Anti-CCP, anti-citrullinated cyclic peptide antibodies; BRM, biologic response modifier; DMARD, disease-modifying anti-rheumatic drug; DAS28, 28-joint disease activity score; MCP, metacarpophalangeal joint; MRI, magnetic resonance imaging.

The study is a three-group, double-blind, randomized controlled trial (Figure 1). The interventions include 1.0 T extremity MRI of the second to fifth MCPs of the worst-affected hand (determined by cumulative swollen and tender joint count at baseline) and X-ray of both hands using lateral, anteroposterior, and oblique views. The two interventions will be superimposed on standard of care. All groups, including the sham-intervention group, will receive standard of care diagnostic imaging feedback. Participants will be assigned to one of the three interventions using minimization, also known as baseline adaptive randomization [41,42]. The prognostic factors for intervention allocation were determined from recent American College of Rheumatology anti-rheumatic treatment recommendations [43]. Intervention allocation will be concealed by having all study participants undertake both diagnostic imaging scans at all time points: one-third will have positive or negative reports of disease progression reported back to the rheumatologist based on MRI scores; one-third will have X-ray-based results reported; and one-third will always have negative reports returned to the rheumatologist. Study rheumatologists are informed of the study protocol. They are aware that one-third of the negative results will not be based on imaging findings and are allowed to prescribe X-ray diagnostic imaging as part of standard of care. All image sets will be anonymized per assessment and scored independently by one of four radiologists using the van der Heijde-modified Sharp Score (vdHSS) for X-ray [44-46] and a non-contrast modification [47] of the OMERACT RAMRIS for MRI [24]. At the beginning of the study, and mid-way through follow-up, a sample of nine image sets for each modality will be read by all four radiologists to determine the inter-rater smallest detectable difference (SDD).

The SDD in imaging scores will be used to adjust for inter-rater reliability in the diagnostic imaging scores. Differences in SDD-adjusted scores will be used to determine disease progression. The analyst determining the SDD will be blinded to patient intervention allocation. Intervention allocation will be concealed from treating rheumatologists by reporting disease progression as changes in SDD magnitudes with follow-up disclosure of neither images, nor imaging features. The selection of the anatomy to compare and the frequency of intervention was based on the following considerations: anatomy most commonly scanned over follow-up in this population [40], least anatomy required to visualize greatest change in disease progression [14,15], and minimization of both radiation exposure for X-ray and MRI scan time.

### Population

Adults with established RA and UIA will be recruited for the study. Subjects fulfilling the Emery and colleagues (2002) early referral to rheumatology recommendation for RA will be included: at least three swollen joints, positive squeeze test for either the MCPs or metatarsophalangeal joints, or at least 30 minutes of self-reported morning stiffness [48]. These criteria are strongly associated with rheumatologic opinion on early IA and strongly associated with DMARD treatment decision-making [49]. Eligible subjects will be at least 18 years of age and have a minimum of 6 weeks of self-reported symptom duration at enrolment.

Subjects fulfilling any of the following criteria will be excluded: history of juvenile idiopathic (or inflammatory) arthritis; rheumatologist-confirmed primary clinical diagnosis of viral arthritis or spondyloarthropathy (ankylosing spondylitis, psoriatic arthritis, reactive arthritis, or inflammatory bowel disease); at the investigator’s discretion, a concomitant condition with medical priority over IA; a concomitant condition that contraindicates treatment with DMARDs (not including sulfa allergy or medically controlled non-terminal liver disease); psychological deficit or diminished capacity to provide independent, informed consent; planned event that prevents study follow-up; extremity MRI scanner contraindications including some joint replacements, stents, pace-makers, neurostimulators, or other metal or electronic implants; other irremovable metallic foreign bodies with magnetic properties or known to interfere with MRI; and a current or historical chronic or high exposure to iron materials.

Study participants will be recruited from the practices of six participating rheumatologists at McMaster University, Hamilton, ON, Canada. The study investigators will prescreen the patients for eligibility and refer them to study staff for the provision of informed consent and enrolment.

### Interventions

Study measures are captured in the schedule of assessments (Table 1). Study participants will attend study visits at weeks 0, 13, 26, 39, 52, 78, and 104. All participants will have MRI and X-ray scans within 4 weeks of biannual study visits from weeks 0 to 104. Clinical assessments, prognosis, and response to pharmacologic treatment will be assessed quarterly during the first year and biannually in the second. Study participants will be accrued over 1 year and followed for 2. At week 0 (baseline) and 104 weeks, all study participants will have an MRI conducted for the second to fifth MCPs of both hands. All participants will also have X-rays of both hands and feet conducted at baseline and 104 weeks.

**Table 1:** Study schedule of assessments

**Assessment**	**Week**						
	**0**	**13**	**26**	**39**	**52**	**78**	**104**
Informed consent	X	X	X	X	X	X	X
OHIP number (each visit)	X	-	-	-	-	-	-
Demographics							
Date of birth	X	-	-	-	-	-	-
Gender	X	-	-	-	-	-	-
Race/ethnicity	X	-	-	-	-	-	-
Dominant hand	X	-	-	-	-	-	-
Household income (socioeconomic status)	X	-	-	-	-	-	-
Pack-years smoked	X	-	-	-	-	-	-
Medical history and comorbidities							
Surgeries	X	X	X	X	X	X	X
Conditions	X	X	X	X	X	X	X
Medications	X	X	X	X	X	X	X
Clinical assessment	X	X	X	X	X	X	X
Symptom duration	X	-	-	-	-	-	-
Morning stiffness duration	X	X	X	X	X	X	X
Swollen joint count	X	X	X	X	X	X	X
Tender joint count	X	X	X	X	X	X	X
Extra-articular features	X	X	X	X	X	X	X
Fatigue	X	X	X	X	X	X	X
Pain – Visual Analog Scale	X	X	X	X	X	X	X
Global physician impression of disease progression	X	X	X	X	X	X	X
Global patient impression of disease progression	X	X	X	X	X	X	X
Diagnostic imaging							
MRI – MCPs 2-5							
Worst-affected hand	-	-	X	-	X	X	-
Both hands	X	-	-	-	-	-	X
Radiography							
Both hands	X	-	X	-	X	X	X
Both wrists	X	-	X	-	X	X	X
Both feet	X	-	-	-	-	-	X
Laboratory tests							
Rheumatoid factor	X	O	O	O	O	O	O
Anti-citrullinated cyclic peptide antibodies level	X	-	-	-	-	-	-
Erythrocyte sedimentation rate	X	X	X	X	X	X	X
C-reactive protein	X	X	X	X	X	X	X
Other	X	X	X	X	X	X	X
Concomitant medications	X	X	X	X	X	X	X
Adverse events	X	X	X	X	X	X	X
Health-related quality of life							
Health assessment questionnaire	X	-	X	-	X	X	X
Health Utility Index Mark 3 and EQ-5D	X	-	X	-	X	X	X
Health resource utilization	X	-	X	-	X	X	X
Work and leisure time productivity	X	-	X	-	X	X	X

X, assessment conducted; O, assessment conducted only if preceding assessment negative.

EQ-5D, EuroQOL general quality of life instrument; MCP, metacarpophalangeal joint; MRI, magnetic resonance imaging; OHIP, Ontario Health Insurance Plan.

### Non-contrast OMERACT RAMRIS

**Figure 2:**
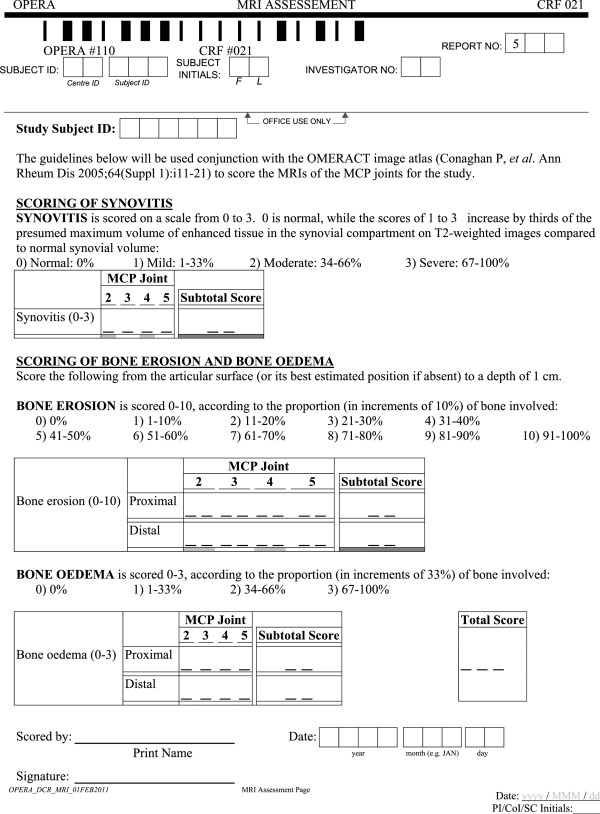
**Outcomes in Rheumatology Rheumatoid Arthritis Magnetic Resonance Imaging Score (OMERACT RAMRIS) form for scoring magnetic resonance imaging (MRI) evidence of disease progression.** MCP, metacarpophalangeal joint.

A non-contrast modification of the OMERACT RAMRIS system will be used to follow disease progression on MRI [47] (Figure 2). The OMERACT RAMRIS is an internationally recognized system for measuring RA disease changes on MRI [24,50-53]. The system is comprised of the following components: a definition of the MRI sequences, planes of view, contrast agent requirements, and signal descriptions for each feature and scoring system [24]; image atlas [50]; guidance on pitfalls with the system [51]; and clinometric property validation studies [52,53]. The non-contrast modification pertains to the measurement of synovitis. In the modification, synovitis is detected on MRI without a contrast agent, using Fast Spin Echo, T2-weighted, fat-saturated sequences [47,54-57]. The OMERACT RAMRIS scale remains the same with the modification.

### van der Heijde-modified Sharp Score

**Figure 3:**
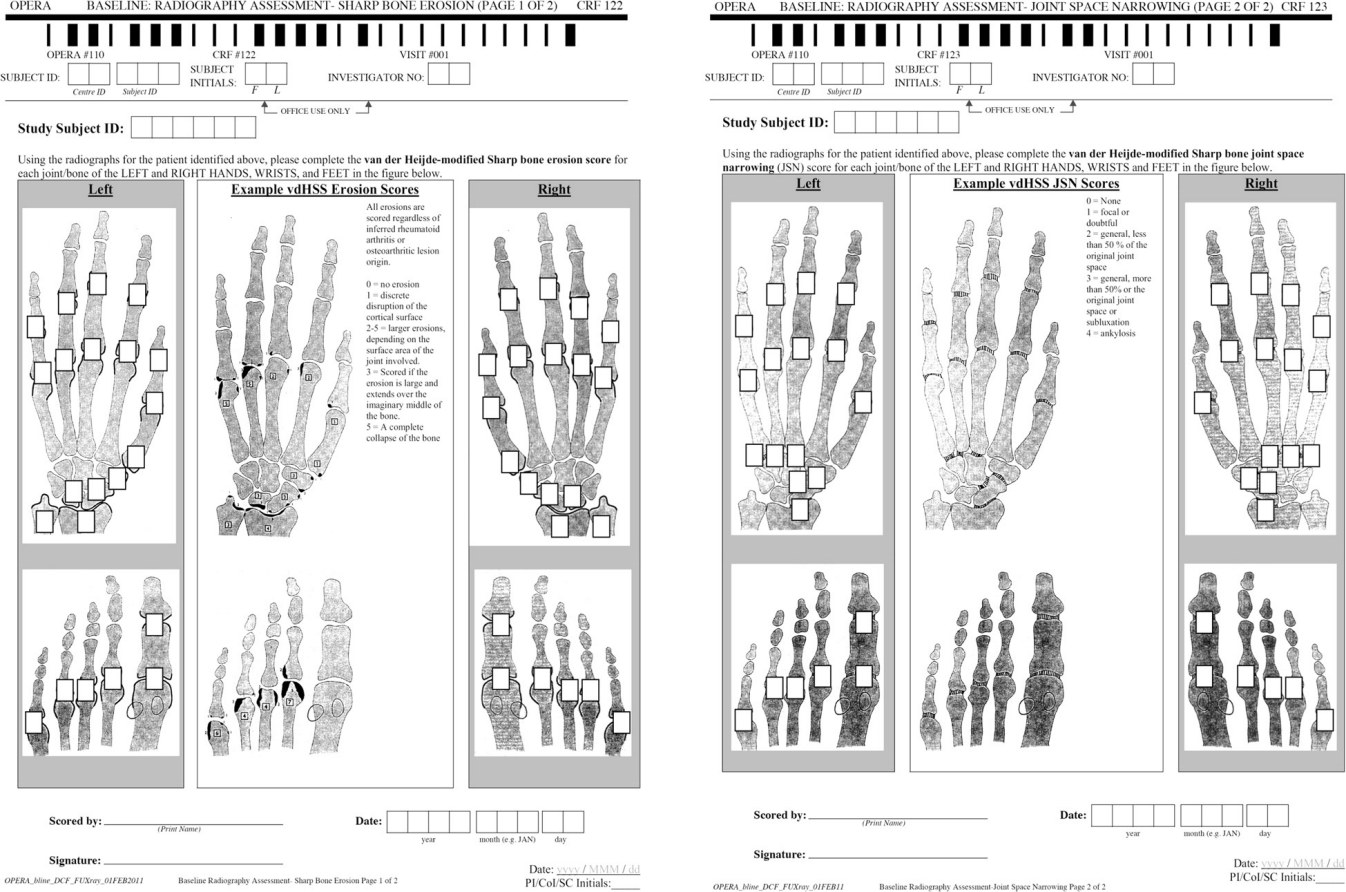
**van der Heijde-modified Sharp Score (vdHSS) forms for scoring X-ray evidence of disease progression.** Left: erosion scoring. Right: joint space narrowing (JSN) scoring.

The vdHSS will be used to measure radiographic disease progression (Figure 3). The scoring system accounts for changes in erosions and JSN on non-continuous, ordinal scales [45,46]. Radiologists will be provided with high-quality printouts of vdHSS assessment examples to guide and promote consistency of scoring over time [45,46] (van der Heijde, personal communication, 2010).

### Diagnostic image scoring

One of four radiologists will score each MRI and X-ray using the modified RAMRIS and vdHSS methods, respectively. Radiologists will be blinded to intervention allocation. The images sets will be anonymized and therefore scored independently from other acquisitions for the same patient. The modified RAMRIS or vdHSS adjusted for inter-rater reliability will be compared between the biannual diagnostic imaging time point and baseline, or to the time point when disease progression was last detected. For week 0 MRI, the modified RAMRIS of the worst-affected hand will be used as the reference score to determine changes. Total baseline tender and swollen joint count will determine the worst-affected hand. If joint counts are equal bilaterally, the participants’ self-reported dominant hand will be used to determine the reference score. For X-ray, the vdHSS for both hands and wrists will be compared biannually to monitor disease progression.

The inter-rater reliability across the four participating radiologists for scoring the RAMRIS and vdHSS, respectively, will be determined using baseline imaging data for the first nine study participants. A similar sample of images sets acquired from patients who first complete the 52-week study imaging requirements will be used to determine changes in SDD over time. The inter-rater SDD determined from the baseline sample will be used to determine adjusted modified RAMRIS and vdHSS scores at baseline and 26-week time points. The SDD determined at the 52-week time point will be used to adjust 52-, 78- and 104-week scores.

Changes in modified RAMRIS and vdHSS scores in magnitudes of SDD will be sent to the treating rheumatologist in the form of blinded diagnostic imaging reports for participants allocated to the MRI and X-ray treatment groups, respectively. For participants allocated to the sham-intervention group, negative reports will be sent. The SDD is a measure of the reliability specific to the raters and procedures of this study [58,59]:

(1) $$\mathrm{SDD}=1.96\times \sqrt{\frac{2\times \mathrm{SE}M_{\mathrm{STATUS}-\mathrm{SCORE}}}{k}}$$

OR

(2) $$\mathrm{SDD}=1.96*\frac{SD_{\mathrm{\Delta STATUS}-\mathrm{SCORE}}}{\sqrt{k}}$$

where *SEM*_*STATUS* − *SCORE*_ is the standard error of measurement of the status-score; *SD*_*ΔSTATUS* − *SCORE*_ is the standard deviation of the difference in status-score; *k* is the number of readings over which the analysis is averaged.

Upon detection of an increase in RAMRIS or vdHSS of at least the SDD, a standardized blinded report will be sent to the rheumatologist indicating the status of disease progression for the patient, as follows:where < # > is the difference of the SDD-adjusted score between the two time points of interest rounded down to the whole number.

“Disease progression < # > -fold the smallest detectable difference was detected on < date of current MRI/X-ray > relative to < date of previous MRI/X-ray>. This disease progression consists of evolving synovitis, and/or edema, and/or joint space narrowing, and/or erosive damage.”

This calculation represents multifold differences in SDD. Disease progression reports sent to the rheumatologist will eliminate references to imaging modality, including reference to either RAMRIS or vdHSS, or of imaging features specific to either modality. These considerations will help maintain allocation concealment.

### Standard of care

All study participants will receive the standard of care for disease management. They will attend clinical assessments quarterly for year 1 and biannually thereafter. Additional clinical visits are allowed per standard of care. The rationale for quarterly visits during the first year is two-fold: a period of sufficient duration to ensure slow-acting DMARDs take effect; a monitoring frequency conducive to detecting treatment adjustments over the early course of care for patients with newly-diagnosed RA or UIA. Pharmacotherapy will be monitored and adjusted over these clinical visits as per rheumatologist-dependent standard of care. Pharmacotherapeutic management of RA and UIA is modified based on monitoring a combination of clinical, laboratory, and diagnostic imaging assessments, in conjunction with patient therapeutic goals. Intra-articular or systemic corticosteroid therapy may be used per standard of care and will be documented. Generally, standard of care may vary by rheumatologist. As a result, the treating rheumatologist is included as a parameter in the minimization treatment allocation scheme.

Study rheumatologists will receive baseline MRI and X-ray reports for all study participants. Upon requisition of extra-study X-ray, the radiology report for the last study-derived images will be made available to the treating rheumatologist per standard of care, regardless of treatment allocation. In contrast, MRI is not standard of care for any of the study investigators and will not be provided. If no progression occurs between diagnostic imaging intervals, or if the participant is allocated to the standard of care intervention arm, negative diagnostic imaging reports will be reported to the treating physician.

As a historical control of standard of care, a chart audit of a randomly selected sample of study-eligible patients visiting the study rheumatologists 3 years preceding study enrolment will be conducted. The chart audit will be used to determine the frequency of diagnostic imaging requisition and treatment escalation. Rates of diagnostic imaging and treatment escalation between the study and historical control will provide a measure of standard of care bias in the study [60] - that is, to determine if either diagnostic imaging or treatment escalation occur more or less frequently within the study than in clinical practice for the same setting.

### Outcomes

#### Primary outcome

The primary outcome of the study is DMARD treatment augmentation over 2 years. All changes in DMARDs and/or biologic treatment over the course of the study will be recorded. The primary endpoint definition is any change in DMARD agent or dose escalation. A co-primary endpoint is diagnostic imaging evidence of disease progression. Clinical radiology reports of the disease progression from discretionary diagnostic imaging requisitions will count towards the assessment of the co-primary endpoint.

#### Secondary outcomes

Secondary outcomes include an alternative categorization of disease-modifying agents (including glucocorticoid therapy), changes in diagnostic imaging (changes in vdHSS, non-contrast modified OMERACT RAMRIS, number of erosions, and proportion of patients with erosions), disease activity (28-joint Disease Activity Score [61], Clinical Disease Activity Index [62], Simplified Disease Activity Index [62], Rheumatoid Arthritis Disease Activity Index [63], Rapid Assessment of Disease Activity in Rheumatology [64], Routine Assessment of Patient Index Data 1-5 [65]), function (Health Assessment Questionnaire [66], Multi-Dimensional Health Assessment Questionnaire [67]), quality of life (Health Utility Index Mark 3 [68], EuroQOL general quality of life instrument [69], EuroQOL general quality of life instrument Visual Analog Scale [69]), healthcare resource utilization and work productivity. Changes from baseline over biannual time points will be investigated. Paired analyses comparing changes in imaging interventions will be conducted. Healthcare resource utilization and work productivity will be determined as adapted from previous work in this population [70]. Healthcare resource utilization will be supplemented by linking patients to provincial healthcare databases. All secondary outcomes are exploratory and therefore hypothesis-generating in nature.

### Randomization

Minimization (that is, baseline adaptive randomization) will be used to allocate participants to one of the three study interventions. Allocation by minimization is recommended for small sample size clinical trials in diseases with many known prognostic markers [41,42]. The minimization procedure can accommodate 10 to 20 variables without sacrificing statistical power [71,72]. Parameters for minimization will include the following:

● Participant self-reported symptom duration (<6, 6-24, >24 months)

● Baseline 28-joint Disease Activity Score (<2.6, 2.6-3.2, 3.2-5.1, >5.1)

● MRI or X-ray evidence of erosions from the hands, wrists or feet at baseline

● Rheumatoid factor positivity (positive; negative; not available)

● Anti- citrullinated cyclic peptide positivity (positive; negative; not available)

● Extra-articular features of RA

● Health Assessment Questionnaire

● Conventional DMARD treatment

● Biologic DMARD treatment

● Treating rheumatologist

With the exception of 'treating rheumatologist', these variables have prognostic value and are expected to inform DMARD treatment decision-making [43]. ‘Treating rheumatologist’ will be included as a minimization parameter to balance the effect of varying pharmacological disease management standards of care by physician across the three study groups.

Minimization will be conducted using a SAS (Cary, NC, USA) macro (Additional file 1). The generic two-group SAS macro for minimization [73] was adapted into the three-group algorithm by Kuznetsova and Dmitrienko (personal communication, March 2011).

### Blinding

Study personnel involved with participant follow-up, care, outcome assessments, or statistical analyses will be blinded to the randomization scheme. Upon determination of eligibility, consent, and value of the minimization parameters for the participant, the enrolling study personnel will place a telephone call to the central study coordinator for the randomization of the participant. Centrally, data entry will be audited in real-time to ensure accuracy. The central study coordinator will assign a randomization number to the participant, record this number on the randomization scheme and report the number to the enrolling study personnel. The caller will record the randomization number in the participant’s case report form.

The MRI and X-ray images will be anonymized, such that direct comparisons between MRI or X-ray images for the same participant and time point, or images for the same participant from the same modality over time cannot be compared. All radiologists’ assessments will be reported back to the unblinded central study coordinator. The central coordinator will then process the results of the radiologists’ assessments based on the intervention allocation of the participants. Blinded disease progression results will be reported back to the study rheumatologist.

### Allocation concealment

The intervention allocation scheme will be concealed from all study participants and study personnel involved in care for participants. Access to the minimization scheme for treatment allocation will be limited to the central unblinded study coordinator or designate. This coordinator will not be involved with the clinical assessment of study subjects or intervention implementation. Statistical analysis will be blinded to treatment allocation.

### Statistical analysis

#### Sample size

A sample size of 186 RA and UIA patients was estimated from a one-way analysis of variance statistical test to determine a difference in the rate of pharmacological treatment escalation between imaging interventions over 2 years between three groups with intermediate dispersion of data [74].

(3) $$f=\frac{d}{2}\sqrt{\frac{\left(k+1\right)}{3\left(k-1\right)}}$$

where *f* is the analysis of variance effect size; *k* is the number of groups being compared; and *d* is the standardized effect size,

(4) $$d=\frac{\delta }{s}$$

where, *δ* is the difference between the greatest and least group mean; and *s* is the standard deviation.

This parametric test is sufficiently robust to yield sample size estimates for non-parametric data [74].

Treatment escalation was assumed to be directly proportional to disease progression. The rate of X-ray progression over 2 years [75] was taken together with 1-year MRI and X-ray progression data [15] to estimate testing between 36% MRI progression of the second to fifth MCPs joints of a single hand, 25% radiographic progression of bilateral hands and wrists, and no progression for the standard of care group. Although some patients may progress several-fold over the clinically relevant threshold, progression was conservatively assumed to result in a single pharmacological treatment escalation per affected participant, since a change in therapy is expected to introduce an incremental protective effect.

The estimated standard deviation around disease progression-related treatment escalation rates was assumed to vary proportionally the variance around disease progression. From Emery and colleagues (2009), an average standard deviation of 5% greater magnitude than the mean progression was determined for group sample sizes of approximately 200 [75]. Standard deviation is inversely related to . A sample size 25% greater than the mean effect size reported in Emery was estimated here. Therefore, *δ* ≈ 0.46 and *s* ≈ 0.58, *f* ≈ 0.33. This represents a sample size of ≈ 45 per group [74] unadjusted for spontaneous remission, attrition and missing data. Despite the liberal inclusion criteria used here, with the current setting characterized by patients with significantly progressed symptom duration, spontaneous remission was conservatively estimated at 5%. Missing data and annual attrition were estimated to be 15% and 5%, respectively. Given these estimates, a sample size of 62 participants per intervention allocation group will be required to determine a 36% difference in the rate of treatment escalation with 90% power at a 5% level of significance [74,76]. A total of 186 IA patients will be recruited by six participating rheumatologists.

### Analysis

Differences in the primary and secondary outcomes between the three groups will be determined using non-parametric tests. Multi-group differences in the rate of pharmacotherapy escalation will be determined using the Kruskal-Wallis test. Differences in the proportion of participants with disease progression will be compared using the Cochran-Mantel-Haenszel test with continuity correction. Two-group differences will be tested using the Wilcoxon Rank Sum (Mann–Whitney U) test. Correction for multiple comparisons will be conducted using the Tukey method.

The primary analysis population will be intent-to-treat. As-treated and per-protocol study populations will also be reported. In the intent-to-treat analysis, the minimization algorithm will be preserved and dropouts will be assumed to have had no progression post-dropout. In the as-treated analysis, patients misallocated relative to the minimization algorithm will be analyzed per the intervention received. In the per-protocol analysis, misallocated participants and other protocol violations specified prior to breaking the blind will be omitted. Complete and Markov chain Monte Carlo multiple imputed data will be reported. The goal of analyzing across different analysis sets is to demonstrate robust study results across the various alternatives. All statistical analyses will be conducted using SAS/STAT version 9.2 (Cary, NC, USA).

### Data management

Data management and monitoring will use iDataFax® (Clinical DataFax Systems Inc., Hamilton, ON, Canada) and SAS/STAT version 9.2.

### Ethics

The study will be conducted in accordance with the principles of the Declaration of Helsinki and has been approved by the St Joseph’s Healthcare Hamilton Research Ethics Board (R.P. #09-3191). Informed consent will be obtained from each study participant.

## Discussion

The primary objective of the study is to investigate the relative value of MRI or X-ray over standard of care as tools to guide DMARD pharmacotherapeutic decision-making by rheumatologists for the care of RA and UIA. It is hypothesized that disease progression determined by MRI or X-ray will result in an increased rate of pharmacotherapeutic escalation. Recent evidence suggests that a proportion of RA patients persist with diagnostic imaging evidence of disease progression while in clinical remission [17].

The potential impact of this study is multifold. First, it will determine if there is value in implementing diagnostic imaging-guided pharmacotherapeutic clinical decision-making at the RA population level or whether further investigation in a targeted subset is warranted. The clinical trial literature is replete with trials demonstrating less than 50% of the population progressing on diagnostic imaging over 2 years [77,78]. However, predicting erosive progression is challenging [79,80]. In the current study, a health economic evaluation will be integrated within the trial and will contribute to the discussion on the value for money of this approach in a sample comprised of RA and UIA participants.

Second, the relative merits of MRI and X-ray as prognosticators in RA and UIA will be determined. The increased sensitivity of MRI for erosions that arise from the direct paired-bone comparisons is evident [14-17,29,30] but must be tempered by the increased false-positive rate for MRI erosions in healthy controls [47,81-83]. When the anatomies commonly imaged in practice are compared, the relative merits are less clear [14,15]. The probability of erosion is uniform neither by patient nor joint. In active disease, substantial erosive changes were noted in many patient joints, while others were spared [84]. These findings set the expectation that the greater number of bones and joints imaged on X-ray may negate the increased sensitivity of MRI in fewer joints. The advantages to imaging a greater number of joints needs to be taken into consideration with the increased demand on patients, cost, and false-positive rate. Further, the features measured for the X-ray and MRI interventions include more than just erosions and differ by modality. In RAMRIS, erosion score accounts for more than 69% of the total potential score [24]. The other MRI features measured are more transient than erosions and account for a minority of the total potential score: synovitis, the most transient, accounts for up to 10% of the total RAMRIS; edema accounts for up to 21% of the total score [24]. In contrast, vdHSS accounts for erosions and JSN. The former accounts 57% of the score for bilateral hands and wrists [45,46]. There is sufficient uncertainty in the relative prognostic merits of MRI and X-ray to argue the position of clinical equipoise in relation to the primary objective.

The merits of the proposed study should to be balanced with its limitations. To this end, the study population may be less than ideal. Challenges exist to adjusting diagnostic imaging evidence of disease progression for measurement reliability. The data used to estimate the sample size requirements were limiting. Other limitations may exist as well. The implications of these limitations and alternatives are discussed below.

First, the study population includes patients with varying degrees of disease activity. This may cause background noise to the signal of the intervention. The evidence that 19% of RA patients in clinical remission progress on diagnostic imaging over 1 year [17] may be limited to a small proportion of patients in clinical practice with quiescent disease. For the remainder of patients with active disease, treatment decision-making based on standard of care disease activity findings may be sufficient. In the presence of clinically active disease, attempts to link the effect of diagnostic imaging evidence of disease progression on treatment escalation may be confounded. The signal from the intervention may be dampened by the noise associated with standard of care treatment escalation. Ideally the study population would be characterized by a treat-to-target standard of care and limited to patients with low disease activity or in clinical remission to minimize the effect of standard of care on treatment escalation. In this trial setting, where the clinical practice use of disease activity measures is limited [40], the potential impact of diagnostic imaging guided care may be magnified.

Second, the application of the SDD has some limitations. In the current study, the inter-rater SDD will be used for simplicity. The SDD was used given that all image sets are to be scored independently. With the well characterized scoring systems used, the independent scoring of individual image sets carries less potential for bias from sources both known and unknown. The SDD will also be assessed at two separate time points to account for changes in inter-rater reliability and hence SDD over time. The approach does not account for intra-rater reliability. In addition, the lack of pairing image sets for the same patient may result in less sensitivity to change compared to alternatives. The smallest detectable change (SDC) is an alternative approach that uses paired images to assess reliability and change. Use of the SDC produces less variability, enabling smaller differences in disease progression to be detected [60]. Implementation of the SDC also carries incremental logistical complexity (for coordinating pairing) and rater scoring time. Acknowledging these tradeoffs, the SDD was proposed for the current investigation.

Third, for the sample size calculation, rates of disease progression were conservatively estimated from literature reports of disease progression between MRI and X-ray. Standard of care treatment escalation and a clinically meaningful effect size were not specifically accounted for in the estimate. Despite this limitation, the authors considered the sample size to be sufficient to resolve any incremental benefit of the interventions in the study population investigated.

A number of factors are expected to impact the relative merits of MRI and X-ray as prognostic tools in IA, including those affecting image resolution (for example, magnet strength to bore diameter ratio for MRI; number of views for radiography) and stage of disease. Measurement considerations including number of raters involved, their qualification and experience, number of readings and raters per image set, independent versus paired image rating, blinding the chronological order of image acquisitions, and current anti-rheumatic therapy are expected to affect the MRI and X-ray interventions equally.

This study represents an initial investigation into the clinical utility of MRI and X-ray in guiding anti-rheumatic pharmacotherapeutic decision-making for IA. The work will contribute to the knowledge of how diagnostic imaging may be leveraged to optimize rheumatologic care. Pending findings, future work will investigate the optimization of this intervention strategy, including but not limited to identifying population subsets at greatest risk, adjusting the interval over which imaging is conducted, testing in conjunction with a specific intensive DMARD clinical management strategy, and automated abnormality detection and quantification.

## Trial status

Ongoing.

## Abbreviations

DMARD: disease-modifying anti-rheumatic drug; IA: inflammatory arthritis; JSN: joint space narrowing; MCP: metacarpophalangeal joint; MRI: magnetic resonance imaging; OMERACT: Outcomes in Rheumatology (formerly Outcome Measures for the Evaluation of Rheumatoid Arthritis Clinical Trials); RA: rheumatoid arthritis; RAMRIS: Rheumatoid Arthritis Magnetic Resonance Imaging Score; SDC: smallest detectable change; SDD: smallest detectable difference; UIA: undifferentiated inflammatory arthritis; vdHSS: van der Heijde-modified Sharp Score.

## Competing interests

The authors declare that they have no competing interests.

## Authors’ contributions

All authors contributed to the study design, analysis plan, and manuscript drafting and approval (CEW’s posthumous authorship precluded manuscript approval). The core study design and analysis plan team include RT, KAB, RG, MJL, JET, CEW and JDA. RT, WGB, RSB, ML, LEH, AAC, NP, KF, EJ and JDA are involved in data collection. RT, RG and JET are involved in data management.

## Supplementary Material

Additional file 1 **Generic SAS (Cary, NC, USA) macro for three-group Pocock and Simon-based minimization intervention treatment allocation **[42]**.** Click here for file
