# Supplementary material for: A double-blind, randomized controlled trial to compare the effect of biannual peripheral magnetic resonance imaging, radiography and standard of care disease progression monitoring on pharmacotherapeutic escalation in rheumatoid and undifferentiated inflammatory arthritis: study protocol for a randomized controlled trial
Source: Trials. 2014 Jul 5;15:268. doi: 10.1186/1745-6215-15-268 (PMC4227117; doi:10.1186/1745-6215-15-268)
Supplement: Additional file 1 — Generic SAS (Cary, NC, USA) macro for three-group Pocock and Simon-based minimization intervention treatment allocation [42]. [file 1745-6215-15-268-S1.pdf]

```
* Macro that executes k-arm minimization;
* A random vector of covariates is generated for testing purposes;
* In a real trial, the vector of covariates of the patient in generation GEN should be read from the real one-observation dataset;
options nomprint;
%macro mink[n=,                          /*the number of patients to be allocated*/
            k=3,                         /*number of treatment arms*/
            nfact=2,                     /*number of factors minimization baalnces upon*/
            lev1=2,                      /*number of levels of teh first factor*/
            lev2=2,                      /*number of levels of the second factor, add more macr variables Levi if NFACT>2*/
            pnon=0.1,                    /*probability to allocate non-preferred treatment*/
            mseed=1971,                  /*random seed*/
            outdata=                     /*the dataset to store the complete allocation log*/];
*0. generate and store the dataset with the random string of a uniformly distributed variable;
data uni;   do gen=1 to &n;    ranu=ranuni[&mseed];    output;    end;    run;
*proc print data=uni;*run;
*1. generate the sequence of covariates for testing purposes modify code for  allocation in a real trial;
data covs;  n=&n;    do i=1 to n;    gen=i;    %do f=1 %to &nfact;    rand&f=ranuni[3171];
            %do lev=1 %to &&lev&f;    c&f._&lev=[ceil[rand&f*&&lev&f]=&lev];
            %end;    %end;    output;    end;    run;
proc print data=covs;run;
* 2. initiate the treatment totals;
data ttotals;    * marginal totals for each treatment;
%do i=1 %to &k;             %do f=1 %to &nfact;    %do lev=1 %to &&lev&f;
            t&i._&f._&lev=0;                        *treatment I total for level LEV of the factor F;
            %end;    %end;    %end;    run;
* initiate dataset to store all allocation records with complete info;
data store;gen=0;run;
proc print data=ttotals;run;
* 3. assign the treatments;
%do gen=1 %to &n;
data assign;    merge covs[where=[gen=&gen] /*covariates of the AN=gen are merged in, edit for real trial*/
            keep=gen    %do f=1 %to &nfact;    %do lev=1 %to &&lev&f;    c&f._&lev
            %end;    %end; ]
ttotals uni[where=[gen=&gen]];                                          *merge in uniform random variable ranu;
            k=&k;    %do i=1 %to &k;
*calculate the imbalance function assuming that the patient is assigned TRT I;
* imbalance is the sum of the marginal ranges of the number of treatments across the factor levels of the patient in generation &gen;
            %do f=1 %to &nfact;    %do lev=1 %to &&lev&f;    if c&f._&lev=1 then do;    %do j=1 %to &k;
            %if &j=&i %then %do;    if&i._&f._&j=t&j._&f._&lev+1;
                                    *treatment I total in the new patient level of Factor F assuming I is allocated;
%end;    %else %do;    if&i._&f._&j=t&j._&f._&lev;
                                                *other treatments total in the new patient level of Factor F;
%end;    %end;    end;    %end;
*now all treatment totals if.. assuming I is assigned are derived ;
*derive the range of trts within the factor F if TRT I is assigned;
            rg&i._&f=range[of if&i._&f._1-if&i._&f._&k];
*derive sum of ranges across all factors - total imbalance for group I;
            totrg&i=sum[of rg&i._1-rg&i._&nfact];    %end;    %end;
*derive minimum of total imbalances across k groups;
            mi=min[of totrg1-totrg&k];
* derive w - number of treatment groups that have total imbalance equal to mi;
            w=0;    %do i=1 %to &k;    if totrg&i=mi then w=w+1;    %end;
if w=k then do;                         *if all groups have equal total imbalance, they are allocated in equal ratio ;
            %do i=1 %to &k;    p&i=1/k;    %end;    end;    else do;
*if W groups have total imbalance equal to minimum total imbalance MI [preferred groups], each of them are allocated with probability [1-&pnon]/w.  The remaining [K-W] non-preferred groups are allocated with probability &pnon/[k-w] each;
            %do i=1 %to &k;    if totrg&i=mi then p&i=[1-&pnon]/w;    else p&i=&pnon/[k-w];    %end;    end;
*choose treatment TRT out of 1-K with probabilities p1-pk using uniform random variable RANU from the merged in dataset UNI;
if ranu le p1  then trt=1;else    %do i=2 %to &k;    if ranu le sum[of p1-p&i] then trt=&i;else    %end;;
*update the treatment totals with NEWTOTALS ;
            %do i=1 %to &k;    if &i=trt then do;%do f=1 %to &nfact;    %do lev=1 %to &&lev&f;
                        if c&f._&lev=1  then t&i._&f._&lev=t&i._&f._&lev+1;
*assigned treatment totals are increased by 1  for levels of the Subject GEN;
            %end;%end;end;%end;
*derive the treatment totals;
            %do i=1 %to &k;    tot&i=sum[of t&i._1_1-t&i._1_&lev1];    %end;    run;
*proc print data=assign;*title "gen=&gen"; *run;
* 4. update the dataset ttotals;
data ttotals;                                           * marginal totals for each treatment;
            set assign;
            keep    %do i=1 %to &k;    %do f=1 %to &nfact;    %do lev=1 %to &&lev&f;    t&i._&f._&lev
                    %end;%end;%end;;    run;
*proc print data=ttotals;*title "gen=&gen"; *run;
*5. append ASSIGN to randomiztaion log;
data store;set store assign;if gen>0;run;
*proc print data=store;*title "gen=&gen"; *run;
%end;                                                   *end of a single allocation sequence generation;
data &outdata;set store;run;                            *complete allocation log;
proc print data=&outdata;    title 'randomization log';    run;
%mend mink; %mink[n=30, k=3,nfact=2, lev1=2, lev2=2, pnon=0.1,mseed=1971, outdata=test1];
```
